# Study on the Effect of Flexible Passive Deformation of Tuna Caudal Fin on Swimming Performance

**DOI:** 10.3390/biomimetics9110669

**Published:** 2024-11-01

**Authors:** Shi-Yun Zhong, Tian-Yu Gao, Wei-Pei Huang, Peng-Nan Sun, Chao Yu, Wang Zhao, Zhi-Qun Guo

**Affiliations:** 1 School of Ocean Engineering and Technology, Sun Yat-sen University, Zhuhai 519082, China; zhongshy7@mail2.sysu.edu.cn (S.-Y.Z.); gaotianyu_mech@163.com (T.-Y.G.); huangwp28@mail2.sysu.edu.cn (W.-P.H.); sunpn@mail.sysu.edu.cn (P.-N.S.); 2 Southern Marine Science and Engineering Guangdong Laboratory (Zhuhai), Zhuhai 519080, China; 3 Beijing Electro-Mechanical Engineering Institute, Beijing 100074, China; yuchaohust@163.com (C.Y.); zhaowang54@163.com (W.Z.)

**Keywords:** caudal fin, passive deformation, vortex dynamics, Strouhal number, propulsion performance

## Abstract

The high-speed and efficient swimming characteristics of tuna are valuable for designing bio-inspired underwater vehicles. Tuna use their highly deformable caudal fins as propulsors during swimming. Caudal fin deformation is categorized into skeletal-controlled active deformation and fluid-induced flexible passive deformation. To investigate how flexible passive deformation affects propulsion performance, simulations of four caudal fins with varying flexibilities under two St numbers in a uniform flow are conducted using the finite volume method. This study finds that the medium-flexibility caudal fin achieves a higher time-averaged thrust coefficient without sacrificing efficiency under both high and low St numbers. At a high St number, the medium-flexibility caudal fin enhances thrust by reducing detrimental secondary flows. At a low St number, the medium-flexibility caudal fin increases thrust by strengthening vortex rings, which induces a stronger backward jet.

## 1. Introduction

Over millions of years, fish have evolved exceptional swimming capabilities. Tuna, as a typical representative, can reach speeds of up to 160 km per hour. Their swimming range spans thousands of kilometers, earning them the nickname “borderless fish”. The high-speed and efficient swimming abilities of tuna are valuable for the design of bionic underwater vehicles, making them a subject of interest for researchers.

Generally, fish swimming modes are divided into two categories: body and/or caudal fin (BCF) mode, and median and/or paired fin (MPF) mode [1,2,3,4]. BCF propulsion can be further divided into five types: anguilliform, sub-carangiform, carangiform, thunniform, and ostraciform [5]. In thunniform swimmers, such as tuna investigated in this paper, significant lateral undulation occurs in the posterior third of the body, with the maximum amplitude at the tail tip [6].

Since the late 20th century, there has been extensive research on tuna and thunniform propulsion. Dewar and Graham [7] conducted a detailed study on the swimming motion characteristics of yellowfin tuna, a thunniform species. Their results indicate that the propulsion wavelength of tuna is 1.23 to 1.29 times the body length, much longer than that of other cruising-adapted bony fish like salmon. Triantafyllou and Triantafyllou [8] constructed a robotic tuna to visualize the wake vortices of tuna during swimming. Donley and Dickson [9] compared the swimming characteristics of mackerel in the carangiform mode and tuna in the thunniform mode, reporting that tuna exhibit a significantly higher tail beat frequency and a significantly lower tail beat amplitude compared to mackerel at a given swimming speed. Donley et al. [10] examined the swimming kinematics of lamnid sharks and demonstrated significant evolutionary convergence between lamnid sharks and tuna.

The caudal fin, as the primary propulsor in thunniform species, generates over 90% of the thrust [11]. Hence, it is necessary to investigate the impact of caudal fin deformation on the swimming performance of tuna. Chopra and Kambe [12] applied lifting surface theory to analyze the propulsive performance of lunate caudal fins in fast marine animals, emphasizing that the thrust and efficiency of the caudal fin depend on the aspect ratio, frequency, and other movement parameters. Adkins and Yan [13] used user-defined functions in Fluent to simulate three-dimensional caudal fin motion behind the fish body and observed the flow behavior. The effect of caudal fin shapes on thunniform swimming performance has been investigated using various approaches [14,15,16], all of which lead to the conclusion that the lunate caudal fins of tuna exhibit the highest efficiency. Shelton et al. [17] found that increasing the frequency and stiffness of oscillating flexible hydrofoils resulted in faster swimming speeds and greater thrust. Feilich and Lauder [18] found that the shape of the caudal fin that performs best varies with different motion parameters. Liu et al. [19] numerically analyzed the hydrodynamic performance and thrust generation mechanism of the oscillating caudal fin, particularly their connection to the structure and evolution of the wake vortex system. Vignesh et al. [20] designed a thunniform fish-type bionic underwater vehicle by mimicking a tuna fish with a lunate caudal fin and numerically examined the interaction between the vehicle’s body and fin.

Although numerous studies have examined caudal fin propulsion, they frequently employ rigid airfoils exhibiting pitch and heave motions. However, when assessing the propulsion performance of the caudal fin, the passive deformation due to fluid forces, alongside the fin’s active motion, is equally significant. Hua et al. [21] conducted a numerical study on the propulsion process of a two-dimensional flexible plate moving in a quiescent fluid and found that appropriate bending stiffness helps the plate achieve optimal propulsion performance. Other studies have also simplified caudal fins into beams or plates, incorporating basic structural constitutive relations and coupling fluid dynamics equations with structural motion equations [22,23,24,25,26]. Zhou et al. [27] superimposed spanwise deformation on the original pitch and heave motions of the caudal fin and briefly investigated the impact of spanwise deformation on thrust and efficiency. Huang et al. [28] utilized the immersed boundary method to conduct a hydrodynamic simulation of a flexible fish carapace, indicating that an increase in carapace height favors thrust generation. Wang et al. [29] investigated the impact of cupped deformation on the hydrodynamics of the caudal fin, emphasizing how the cupped phase difference affects hydrodynamic performance under various Reynolds numbers (Re) and Strouhal numbers (St).

Previous studies typically simplify the caudal fin as beams and plates, overlooking its lunate shape. Systematic research on the impact of passive deformation on the propulsion performance of the tuna caudal fin is lacking. Nevertheless, the issue of passive deformation is complex, and its modeling is challenging. Moreover, natural passive deformation is uncontrollable. Therefore, this paper introduces an imposed motion to the original model. This approach allows for a more intuitive investigation into the impact of passive deformation and facilitates its adoption in engineering applications.

The paper is arranged as follows. Firstly, the motion model of the caudal fin and related numerical methods are introduced in Section 2. Based on these, the results and a discussion are provided in Section 3. Finally, conclusions are provided in Section 4.

## 2. Materials and Methods

### 2.1. Geometrical Model

**Figure 1:**
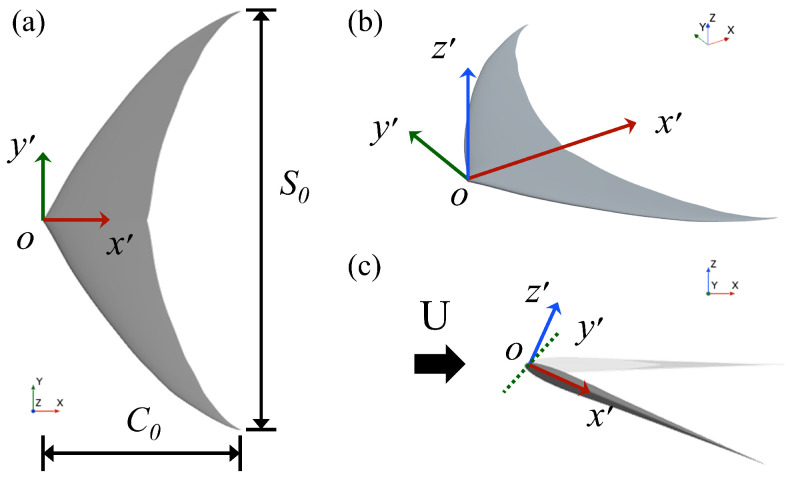
The configuration of the caudal fin model: (**a**) The caudal fin model. (**b**) The body-fixed coordinate system of the caudal fin. (**c**) The diagram of fin motion.

The shape of the passively deforming caudal fin in this paper is based on the work by Liu et al. [19], as shown in Figure 1. A body-fixed coordinate system is positioned at the leading edge vertex of the caudal fin, with the chordwise direction as the $x^{\prime }$-axis and the spanwise direction as the $y^{\prime }$-axis. The caudal fin is set in the steady flow with constant speed *U*. $C_{0}$ is the chord length, $S_{0}$ is the span length and $S_{0}$ = 2$C_{0}$.

### 2.2. Motion and Deformation

The basic motion equations of the caudal fin include heave and pitch, which can be expressed as

(1) $$\{\begin{array}{c} z\left(t\right)=z_{0}sin\left(2\pi ft\right),\\ \theta \left(t\right)=\theta_{0}sin\left(2\pi ft-\pi /2\right), \end{array}$$

where z(t) and θ(t) represent the heave displacement and pitch angle. $z_{0}$ and $\theta_{0}$ are the maximum amplitude of the heave and pitch. *f* is the beat frequency.

**Figure 2:**
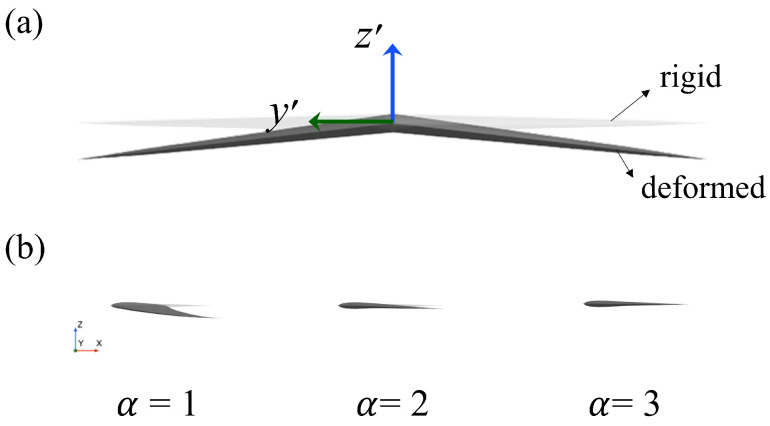
The diagram of passive deformation of the caudal fin: (**a**) Comparison between the rigid fin and passively deforming fin. (**b**) Deformation with various rigidity coefficients.

In addition to the aforementioned motion according to the global coordinate system, the fin is subjected to a spanwise deformation, which can be expressed as

(2) $$z^{\prime }\left(y^{\prime },t\right)=A{\left(\frac{\left|y^{\prime }\right|}{S_{0}/2}\right)}^{\alpha }\sin\left(2\pi ft-\pi \right),$$

where $z^{\prime }\left(y^{\prime },t\right)$ is the total deformation, which is related to the spanwise distance $y^{\prime }$ and time *t*. Here, α is the rigidity coefficient, and $A=S_{0}/4$ is the deformation amplitude. Figure 2 shows the diagram of flexible passive deformation and deformation with different rigidity coefficients.

The Reynolds number (Re) and the Strouhal number (St) for the caudal fin in the present work are defined as [19]

(3) $$Re=\frac{UC_{0}}{\upsilon },$$

and

(4) $$St=\frac{2z_{0}f}{U},$$

where υ is the kinematic viscosity of the fluid.

The dimensionless force coefficients for thrust ($F_{T}$), lift ($F_{L}$), and the moment about the $y^{\prime }$-axis ($M_{y^{\prime }}$) of the caudal fin are expressed as

(5) $$\{\begin{array}{c} C_{T,L}=\frac{F_{T,L}}{0.5\rho C_{0}S_{0}U^{2}},\\ C_{M}=\frac{M_{y^{\prime }}}{0.5\rho C_{0}^{2}S_{0}U^{2}}, \end{array}$$

where ρ = 1000 kg/m^3^ is the fluid density and *U* is the inflow velocity.

According to the propulsion motion Equation (1), the time-averaged input power is defined as

(6) $$\;P_{in}=\frac{1}{nT}\int_{0}^{nT}\left[F_{L}\frac{dz}{dt}+M_{y^{\prime }}\frac{d\theta }{dt}\right]dt,$$

The time-averaged output power, also known as the useful work, is defined as

(7) $$P_{o}=\frac{1}{nT}\int_{0}^{nT}F_{T}Udt.$$

Thus, the efficiency of caudal fin is obtained as follows:

(8) $$\eta =\frac{P_{o}}{P_{in}}.$$

### 2.3. Numerical Method

In this paper, simulations are conducted by the commercial software Star-CCM+, which is based on the finite volume method. The Reynolds-averaged Navier–Stokes (RANS) model is employed, which can be written as [30]

(9) $$\{\begin{array}{c} \frac{\partial \rho }{\partial t}+\frac{\partial \left(\rho \bar{v_{i}}\right)}{\partial x_{j}}=0,\\ \frac{\partial \left(\rho \bar{v_{i}}\right)}{\partial t}+\frac{\partial \left(\rho \bar{v_{i}v_{j}}\right)}{\partial x_{j}}=-\frac{\partial \bar{p}}{\partial x_{i}}+\frac{\partial }{\partial x_{j}}\left(\mu \frac{\partial \bar{v_{i}}}{x_{j}}-\rho \bar{v_{i}^{\prime }v_{j}^{\prime }}\right)+S,\left(i,j=1,2,3\right), \end{array}$$

where ρ is fluid density, *v* is velocity, *p* is pressure and μ is viscosity. The terms $\bar{v}$ and $\bar{p}$ represent the time-averaged components, and $v^{\prime }$ represents the fluctuating component. *i* and *j* refer to the *i*-th or *j*-th Cartesian direction. *S* represents the additional forces or energy sources.

**Figure 3:**
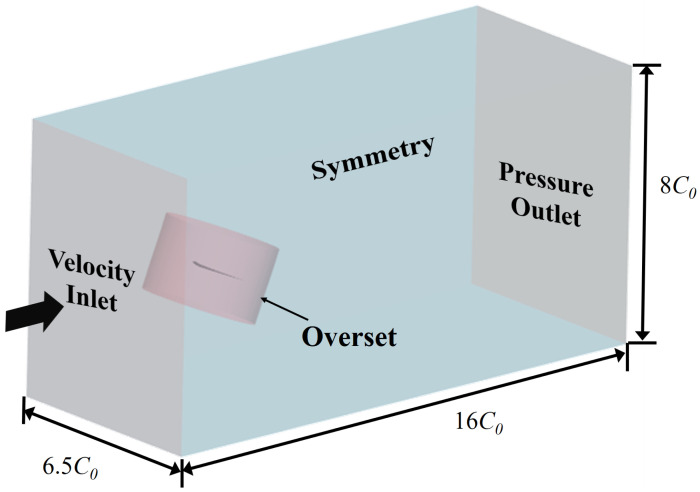
The configuration of the computational domain.

The pressure–velocity coupling is solved using the SIMPLE algorithm. Meanwhile, time integration is handled using a second-order accurate implicit solver to ensure high computational precision. For wall treatment, the all-y+ approach is applied, and the fin surface is modeled as a no-slip wall. Furthermore, the SST k−ω turbulence model [31] is employed in this study. Both the overset mesh approach and morphing motion are implemented in simulations. The overset meshes are suitable for problems in dealing with large motions, which are applied to simulate the heave-and-pitch motion of the caudal fin. Meanwhile, imposed deformation motion is performed using morphing based on the Radial Basis Function (RBF). The computational domain, boundary conditions and the overset region are depicted in Figure 3.

### 2.4. Validation

In this section, the accuracy and stability of the present numerical model are validated. Firstly, a benchmark of the rigid fin with $C_{0}$ = 0.1 m referring to Liu et al. [19] and Zhang et al. [14] is employed. In this benchmark, the inflow velocity is *U* = 0.33 m/s, the beat frequency is *f* = 0.5 Hz, the maximum pitch angle is $\theta_{0}$ = 25°, and the maximum heave is $z_{0}$ = 0.1 m. The dimension and kinematic parameters of the benchmark and subsequent cases are displayed in Table 1.

**Figure 4:**
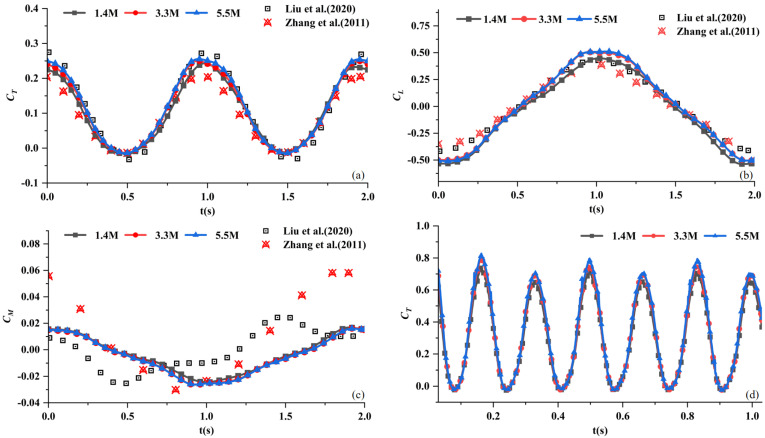
Comparison between numerical results and reference results [14,19] in the benchmark and the grid convergence of a deforming fin: (**a**) Comparison of the thrust coefficient between the present model and reference data. (**b**) Comparison of the lift coefficient between the present model and reference data. (**c**) Comparison of the moment coefficient between the present model and reference data. (**d**) Grid convergence test of the thrust coefficient of the deforming fin with α = 1 and St = 0.6.

In the benchmark validation, three different mesh resolutions are adopted: 1.4 million, 3.3 million, and 5.5 million cells. As shown in the Figure 4a–c, the coefficients of thrust, lift, and moment are compared. The numerical results of the present model show good agreement with reference data, confirming the capability of the current numerical method for modeling the rigid fin.

As for the passively deforming model, we also conducted a convergence test of the thrust coefficient. It should be noted that the current scale for the deforming fin is different. The fin with a span length $S_{0}$ = 0.37 m is employed in all subsequent simulations. The present study focuses on investigating the hydrodynamic performance of a flapping caudal fin under various Strouhal numbers and rigidity coefficients. Therefore, the convergence analysis of the deforming model is conducted under conditions of maximum deformation and most intense flapping, with α = 1 and St = 0.6.

In the convergence analysis of the deforming fin, the inflow velocity was *U* = 2.5 m/s, the beat frequency was *f* = 3 Hz, the maximum pitch angle was $\theta_{0}$ = 22.5°, and the maximum heave was $z_{0}$ = 0.255 m. Three mesh resolutions of 1.4 million, 3.3 million, and 5.5 million cells with time step ΔT = 0.001 s were performed, as shown in Figure 4d. The results demonstrate that higher mesh resolutions led to more accurate predictions, but the improvements became marginal beyond 3.3 million cells. Therefore, the 3.3 million cell mesh was selected, considering both computational resources and computational accuracy.

Through this validation process, the reliability and accuracy of the numerical model were assessed. This evaluation confirmed the capability of the present numerical model to accurately simulate both rigid and deforming fins.

## 3. Results and Discussions

In the following sections, the fin with $S_{0}$ = 0.37 m of different flexibilities is adopted in simulations. Increased flexibility of the caudal fin results in greater passive deformation due to fluid forces. In this context, α denotes the rigidity coefficient. A smaller value of α indicates greater flexibility of the caudal fin. When α approaches infinity, the caudal fin is considered rigid and does not exhibit passive deformation due to fluid forces. The Reynolds number in the simulations is Re=20000.

### 3.1. Case 1: St = 0.6

**Figure 5:**
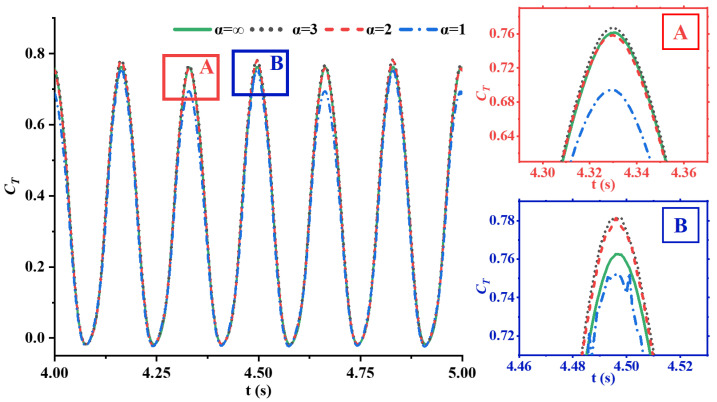
The evolution of the thrust coefficients of four caudal fins with different flexibilities over time at St=0.6, where regions A and B show enlarged views of thrust peaks to highlight differences between various rigidity coefficients.

As is shown in Figure 5, the evolution of the thrust coefficient over time for caudal fins with four different flexibilities is depicted. From Figure 5, it can be observed that the evolution trends of the thrust coefficient curves for caudal fins with different flexibilities are generally consistent. During a single flapping cycle of the caudal fin, the symmetrical up-and-down flapping motion results in two cycles of the thrust coefficient for the four types of flexible caudal fins. During the middle stages of the caudal fin’s upstroke or downstroke, the fin moves at a high flapping speed, generating strong interactions with the fluid, which results in peak thrust coefficients. In contrast, during the stroke reversal phase, the instantaneous flapping speed of the caudal fin is 0, leading to weaker interactions with the fluid, and the thrust coefficient reaches its trough. The moments when the thrust coefficient reaches its peak are almost identical in four cases, indicating that the degree of flexibility, or passive deformation, does not affect the phase of the thrust coefficient curve. Notably, at the thrust peak indicated by the red box (region A), the thrust of the caudal fin with the greatest flexibility is significantly lower than that of the other three fins with less flexibility. The thrust peak values for fins with rigidity coefficients α=2 and α=3 are nearly identical to the thrust peak value of the rigid fin. At the second thrust coefficient peak within the blue box (region B), the thrust peak of the most flexible caudal fin is much lower than that of the others. Furthermore, the fins with moderate flexibility, specifically with α=2 and α=3, exhibit higher thrust peaks compared to the rigid fin, with the thrust peak of the α=3 fin being slightly higher than that of the α=2 fin. Additionally, at the two troughs of the caudal fin’s thrust coefficient, the thrust coefficients of fins with different flexibilities are nearly identical. The results indicate that flexibility significantly affects thrust during the middle stages of the upstroke or downstroke. However, during the stroke reversal phase, changes in flexibility have almost no impact on thrust.

**Figure 6:**
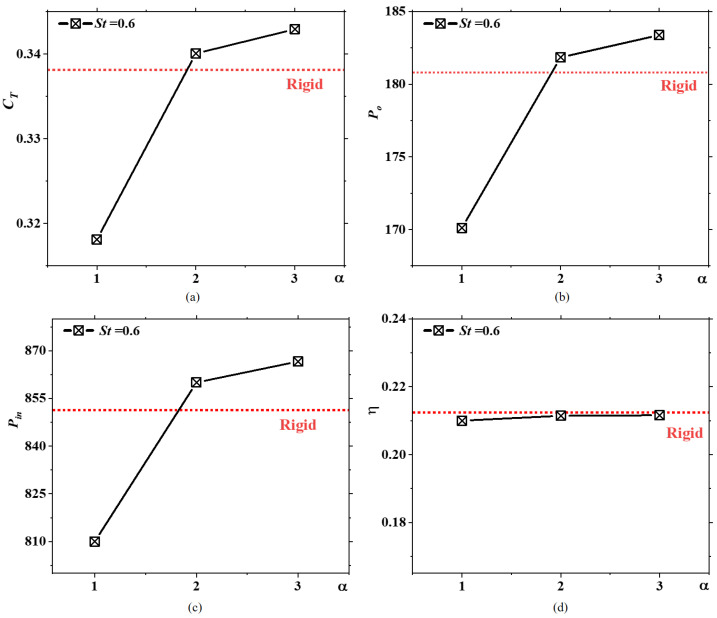
The (**a**) time-averaged thrust coefficients, (**b**) useful power, (**c**) input power, and (**d**) propulsive efficiency of four caudal fins with different flexibilities, with the results for the rigid fin represented by horizontal red dashed lines, at St=0.6.

Figure 6a shows the time-averaged thrust coefficients of four caudal fins with different flexibilities. As the rigidity coefficient α of the caudal fin increases from 1 to 3, the time-averaged thrust coefficient gradually increases, indicating an improvement in the tuna’s cruising speed [32]. Nevertheless, as α approaches infinity and the caudal fin behaves like a rigid material, as in the case represented by the red line, the time-averaged thrust coefficient decreases compared to that of fins with moderate flexibility. Notably, when the caudal fin has maximum flexibility, i.e., α=1, the time-averaged thrust coefficient is actually lower than that of the rigid fin. Figure 5 shows that this reduction is due to the significant attenuation of one of the thrust peaks at α=1. Hence, caudal fins with medium flexibility can achieve maximum thrust, indicating that there is an optimal intermediate rigidity coefficient corresponding to the highest cruising speed for a swimming tuna. This observation aligns with previous studies on self-propelled two-dimensional flexible plates [21]. Hua et al. [21] highlighted that plates with medium flexibility achieve the highest cruising speeds. Figure 6b shows the useful power for propulsion generated by caudal fins with different flexibilities. The variation in useful work carried out by the caudal fin with different rigidity coefficients mirrors the trend in the time-averaged thrust coefficient as rigidity changes. The useful work carried out by the caudal fin increases as the rigidity coefficient α rises from 1 to 3. However, as α approaches infinity, the useful work significantly decreases, yet it remains higher than when α=1. This is because the characteristic velocity is taken as the uniform inflow, making the useful power solely determined by the thrust coefficient. Figure 6c depicts the input power of caudal fins with different flexibilities. It can be observed that the trend in input power with varying rigidity coefficients is consistent with the trend in useful power with rigidity coefficients. Fins with medium flexibility exhibit the highest input power, while the input power for rigid fins exceeds that of fins with maximum flexibility. Therefore, the propulsive efficiency of caudal fins with different flexibilities is nearly identical, as shown in Figure 6d, indicating that the effect of the rigidity coefficient on propulsive efficiency is minimal. The findings from a previous [21] study indicate that for St<1, variations in flexibility over a considerable range do not significantly impact propulsive efficiency. The conclusions presented here align with these findings. In biological propulsion, passive deformation is a double-edged sword. While appropriate passive deformation can be beneficial for thrust generation, excessive deformation should be avoided. In summary, caudal fins with medium flexibility can generate a significant thrust coefficient without reducing efficiency, enabling tuna to maintain higher cruising speeds. This observation also explains why fish caudal fins in nature are always flexible. For biomimetic underwater vehicles, selecting the optimal rigidity coefficient can guide the choice of materials for designing biomimetic caudal fin propellers.

**Figure 7:**
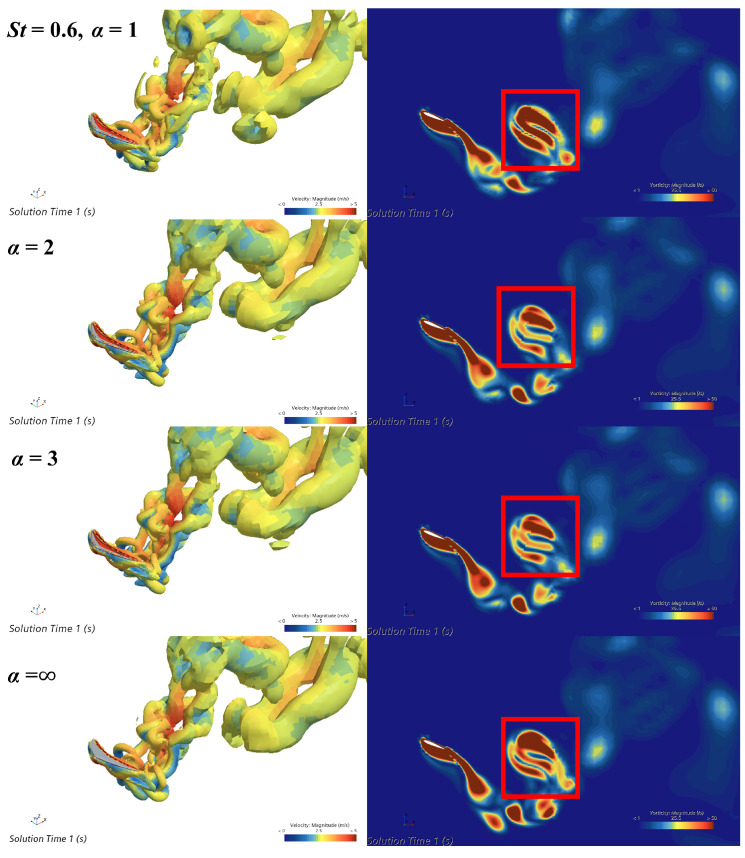
Three-dimensional vortex structures (**left** column) and two-dimensional vorticity magnitude fields (**right** column) of the wakes of four caudal fins with different flexibilities, at St=0.6.

Figure 7 illustrates the three-dimensional vortex structures and two-dimensional vorticity magnitude fields in the wakes of four caudal fins with different flexibilities. The three-dimensional vortex structures of the caudal fins with different flexibilities are quite similar, all exhibiting a double-row vortex ring structure. This is very similar to the wake obtained by Borazjani et al. [33] in simulations of three-dimensional carangiform fish. It is worth noting that the upper and lower rows of vortex rings are notably irregular, containing vortex tubes that extend inward into the vortex street. These tubes indicate complex secondary flows within the wake. In fish swimming, the reverse Kármán vortex street represents the primary vortex structure along the main flow direction, while those small, localized vortices can be seen as disturbance flows that form on top of the main flow. In some cases, they can be considered secondary flow, as their directions do not align with the main vortex structure, and they introduce a certain degree of local irregularity and energy dissipation. The lateral secondary flows arise from the caudal fin’s complex lunate shape and the three-dimensional effects of its flapping. Unlike the backward jet formed by a reverse Kármán vortex street, these secondary flows partially disrupt the wake field and negatively impact thrust generation. In an efficient swimming pattern, fish generate a reverse Kármán vortex street in the fluid through the oscillation of their caudal fin. This vortex street maximizes propulsion performance. However, secondary flow disrupts the orderly structure of this vortex street, preventing the vortices from effectively generating thrust. This disruption leads to discontinuity and instability in thrust, thereby weakening the fish’s swimming ability. Some previous research findings also show that small fragmented vortices caused by secondary flow are detrimental to the propulsion performance of fish [34,35]. The right column of Figure 7, corresponding to the three-dimensional vortex structures in the left column, displays the vorticity magnitude in two-dimensional transverse slices of the wakes from caudal fins with varying flexibilities. The red boxes highlight regions of the previously mentioned secondary flows. When α=1, the high-vorticity region has the largest area, indicating that the most flexible caudal fin generates the strongest secondary flows. These flows reduce thrust generation, resulting in the lowest thrust for the most flexible fin. At α=∞, the high-vorticity region is second in size to that at α=1. Consequently, the thrust generated by the rigid fin is the lowest among the four conditions, except for the most flexible fin. Furthermore, the high-vorticity regions at α=2 and α=3 are almost identical in size and significantly smaller than those at α=0 and α=1. Thus, medium-flexibility fins, at α=2 and α=3, generate the weakest secondary flows, resulting in maximum thrust. These findings align with the time-averaged thrust coefficients in Figure 6.

### 3.2. Case 2: St = 0.27

**Figure 8:**
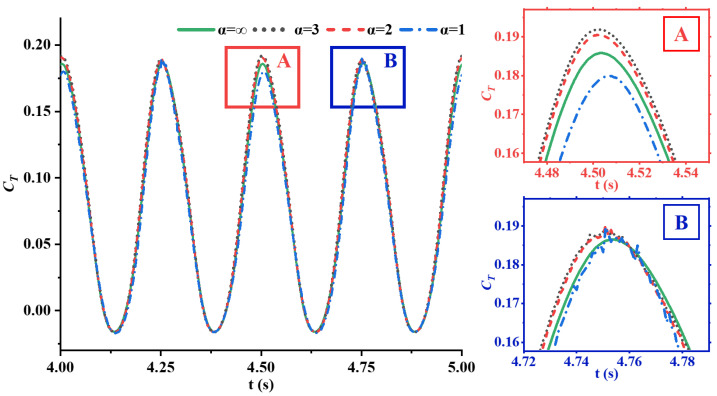
The evolution of the thrust coefficients of four caudal fins with different flexibilities over time at St=0.27, where regions A and B show enlarged views of thrust peaks to highlight differences between various rigidity coefficients.

Figure 8 shows the time history curves of the thrust coefficients for four caudal fins with different flexibilities at St=0.27. Within one flapping cycle, the thrust coefficient exhibits two periods. At the first thrust peak within the red box(region A), the caudal fin with the greatest flexibility has the lowest peak thrust, followed by the rigid fin. The fins with medium flexibility, at α=2 and α=3, achieve the highest peak thrust. At the second peak, indicated by the blue box (region B), the peak thrust coefficients for all four fins with different flexibilities are nearly identical, but for α=2 and α=3 fins, they are still slightly higher than that of the rigid fin. Hence, appropriate flexibility can increase the peak values of the thrust coefficient–time history curve. At the troughs of the thrust coefficient, the trough values obtained by caudal fins with different flexibilities are nearly identical.

**Figure 9:**
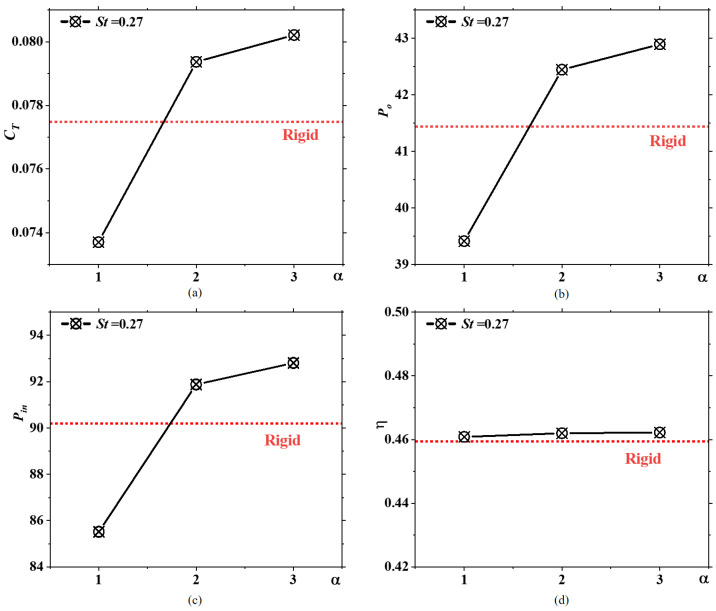
The (**a**) time-averaged thrust coefficients, (**b**) useful power, (**c**) input power, and (**d**) propulsive efficiency of four caudal fins with different flexibilities, with the results for the rigid fin represented by horizontal red dashed lines, at St=0.27.

Figure 9a shows the time-averaged thrust coefficients of caudal fins with different flexibilities, where it can be observed that the fins with α=2 and α=3 acquire higher thrust coefficients. This conclusion is consistent with the findings at *St* = 0.6, further demonstrating that caudal fins with medium flexibility can achieve greater thrust coefficients. Increasing the caudal fin’s flexibility appropriately can ultimately enhance cruising speeds. Figure 9b shows the useful work of four caudal fins with different flexibilities. Since the inflow velocity is constant, the useful work is solely related to the thrust coefficient. Therefore, the trend of useful work with respect to the rigidity coefficient α is consistent with the trend of the time-averaged thrust coefficient with α. As shown in Figure 9c, the input power is highest for the two moderately flexible caudal fins and lowest for the most flexible caudal fin. The input power for the rigid caudal fin is greater than that of the most flexible caudal fin but less than that of the two moderately flexible caudal fins. The trend in input power across varying rigidity coefficients aligns with the trend in useful work. Consequently, the propulsion efficiencies of the four caudal fins with different flexibilities show little variation, essentially aligning along the same horizontal axis in Figure 9d. This conclusion remains consistent with the findings at St=0.6. It can be inferred that the impact of flexibility on propulsion performance is generally consistent across different St values. For various St values, caudal fins with medium flexibility achieve the highest time-averaged thrust coefficients, while fins with high flexibility exhibit even lower thrust coefficients than rigid fins. For caudal fins, appropriate passive deformation can enhance propulsion performance; however, excessive passive deformation can have a negative impact on thrust generation. Within the range of flexibility discussed in this paper, the degree of flexibility has minimal impact on propulsion efficiency. Hence, when designing biomimetic caudal fin propellers, materials with medium flexibility are the most desirable, as they can ensure that the vehicle propels both quickly and efficiently.

**Figure 10:**
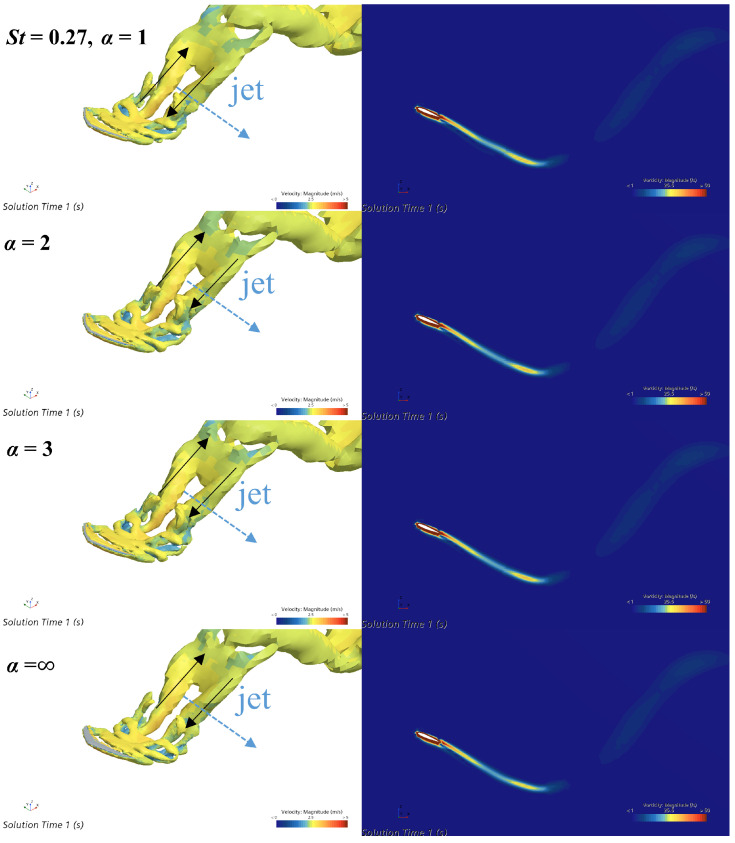
Three-dimensional vortex structures (**left** column) and two-dimensional vorticity magnitude fields (**right** column) of the wakes of four caudal fins with different flexibilities, at St=0.27.

Figure 10 shows the three-dimensional vortex structures in the wakes of four caudal fins with different flexibilities, along with the vorticity magnitude fields on the two-dimensional transverse slices. The left column shows the three-dimensional vortex structures of the wakes. The vortex structures of the four caudal fins with different flexibilities are essentially similar, all forming a typical single-row reverse Kármán vortex street. This single-row vortex street is very similar to the simulation results by Liu et al. [19]. The direction of the vortex tubes on both sides of the vortex rings is indicated by the black arrows. Referring to the analysis method by Kern et al. [36], two vortex lines with different directions induce two vortices with opposing orientations, and these two vortices further induce a backward jet flow. A similar jet flow was also observed in the research findings of Liu et al. [19]. Therefore, a jet is induced in the direction indicated by the blue arrow. According to Newton’s second law, this jet produces a significant forward propulsive force on the caudal fin. It can be observed that when α=2 and α=3, the vortex tubes on both sides of the vortex rings are thicker and visually almost connected. This indicates that the vortex tubes have higher strength, resulting in a stronger backward jet and, consequently, a greater propulsive force for the caudal fin. In contrast, when α=0 and α=∞, the vortex tubes on both sides of the vortex rings are relatively thinner, with a visible gap between them, indicating lower strength and a weaker induced jet. Therefore, the thrust coefficients obtained by the most flexible and rigid caudal fins are relatively low. The higher thrust coefficients achieved by the moderately flexible caudal fins may be related to the higher intensity of the vortex rings they induce. The vorticity magnitude contour plots in the transverse direction for the four caudal fins with different flexibilities are generally consistent, as shown in the right column of Figure 10. This uniformity may be due to the relatively low unsteadiness of the caudal fin’s flapping motion under the present conditions, along with a less pronounced effect of the rigidity coefficient on thrust compared to St=0.6. Consequently, the contour plots do not show very distinct differences.

### 3.3. The Effect of St on the Propulsion Performance of Flexible Caudal Fins

In biomimetic propulsion, St is a crucial dimensionless number that characterizes the degree of unsteadiness in the flapping motion of the biomimetic foil, thereby reflecting the strength of the interaction between the biomimetic foil and the fluid. Furthermore, for the flexible caudal fins addressed in this paper, according to the prescribed passive deformation equation, a larger St will actually result in greater passive deformation. The previous two subsections analyzed the impact of the caudal fin’s rigidity coefficient α on propulsion performance at the same St. Therefore, this subsection examines the effect of St on the propulsion performance of flexible caudal fins.

For comparative purposes, Table 2 provides detailed data on various propulsion performance metrics for caudal fins with differing flexibilities across two St numbers. As can be seen from Table 2, the time-averaged thrust coefficient $\bar{C_{T}}$, useful work $P_{o}$, and input power $P_{in}$ for the four different flexible caudal fins are all greater at St=0.6 compared to St=0.27. This is because, at St=0.6, the increased unsteadiness of the caudal fin’s flapping motion leads to more pronounced interactions with the fluid. When St=0.27, the caudal fin with a rigidity coefficient of α=3 achieves the highest thrust, with an increase of 0.58 N compared to the rigid fin. At St=0.6, the same flexible caudal fin again achieves the highest thrust, with an increase of 1.03 N over the rigid fin, a nearly double increase compared to St=0.27. This indicates that as St increases, the caudal fin undergoes more significant deformation, making the impact of the rigidity coefficient on thrust more pronounced. Notably, the propulsive efficiency of the caudal fin at St=0.27 is significantly greater than at St=0.6, with the efficiency being more than double. This aligns with the widely accepted conclusion that fish typically achieve the highest cruising efficiency when 0.2<St<0.4 [37].

**Figure 11:**
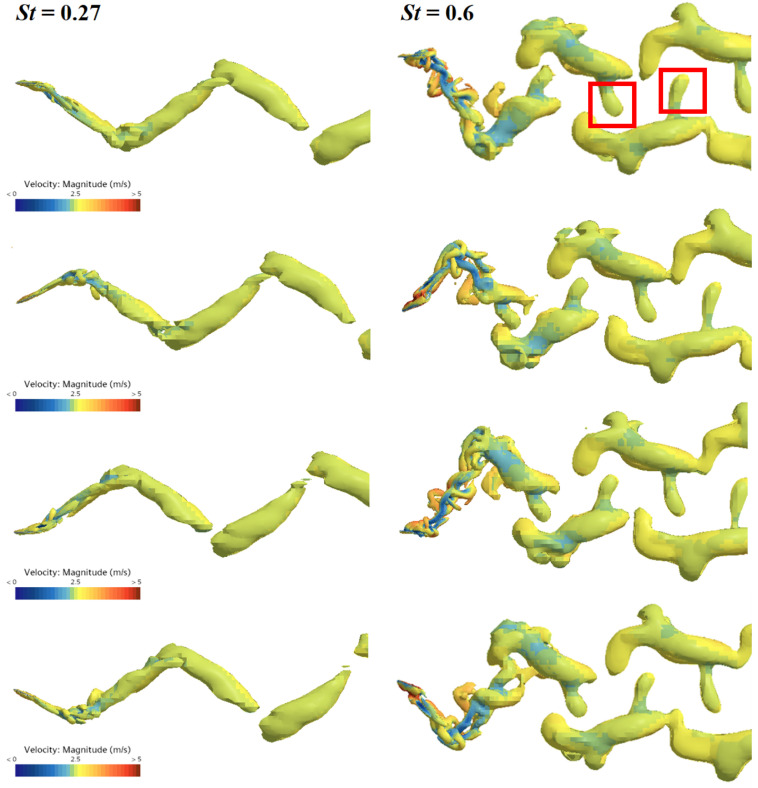
The vortex structure evolution of the caudal fin with a rigidity coefficient of α=3 over one cycle at St=0.27 (**left** column) and St=0.6 (**right** column).

The left column of Figure 11 shows the vortex structure evolution of the flexible caudal fin with α=3 at St=0.27. It can be observed that the vortex structures in the wake form a typical single-row reverse Kármán vortex street, where each vortex ring induces a backward jet. At St=0.6, the vortex street in the wake exhibits a double-row pattern, as shown in the right column of Figure 11. When the flexible caudal fin flaps at a higher St, the wake structure tends to develop from a single-row to a double-row vortex street, consistent with the findings of Borazjani et al. [33]. Additionally, Triantafyllou et al. [38] emphasized that fish achieve optimal propulsion efficiency when the wake behind the body forms a reverse Kármán vortex street, which also explains why the propulsion efficiency of the caudal fin at St=0.27 is approximately twice that at St=0.6. Furthermore, at St=0.6, there is a vortex tube extending inward from the double-row vortex rings in the wake. This vortex tube induces secondary flows that may disturb the wake field, consuming energy and thus impacting the propulsion efficiency of the caudal fin.

## 4. Conclusions

In this study, the flapping motion of four caudal fins with different flexibilities in uniform inflow at two different St numbers is numerically simulated using the finite volume method combined with the overset mesh method. The effect of the caudal fin’s rigidity coefficient, i.e., passive deformation, on propulsion performance metrics such as thrust coefficient and efficiency is analyzed. Additionally, the impact mechanism of passive deformation on the propulsion performance of the caudal fin is examined through vortex structure analysis. The main conclusions are as follows:

- Whether at St=0.27 or St=0.6, caudal fins with medium flexibility achieve higher thrust coefficients. However, the mechanisms for increasing thrust differ at different St values. At St=0.6, medium flexibility enhances thrust by suppressing secondary flows in the wake that are detrimental to thrust generation. At St=0.27, increased thrust is achieved by enhancing the vortex ring structures in the wake, thereby strengthening the backward jet.
- At St=0.27 and St=0.6, the flexibility of the caudal fin has no significant impact on propulsion efficiency.
- At St=0.27, both rigid and flexible caudal fins exhibit much higher propulsion efficiency compared to St=0.6. As St increases, the unsteady effects of passive deformation become more pronounced, making the impact of the rigidity coefficient on propulsion performance more pronounced.

This study suggests that increasing flexibility appropriately can enhance thrust coefficients without compromising propulsion efficiency, enabling higher cruising speeds. These findings provide guidance for designing tuna-inspired caudal fin propulsion systems. Numerical calibration can identify an optimal rigidity coefficient to select materials for caudal fins, achieving optimal propulsion performance. Notably, this study does not examine the impact of flexible deformation on propulsion performance across different Reynolds numbers. Future research will investigate the effects of passive deformation on caudal fin propulsion performance over a wider range of parameters.

## Figures and Tables

**Table 1:** Kinematic parameters of the caudal fin and simulation settings.

Case	$S_{0}$	$z_{0}$	$\theta_{0}$	*f*	St
Benchmark	0.2 m	0.1 m	$25^{\circ }$	0.5 Hz	0.3
Case 1	0.37 m	0.17 m	$22.5^{\circ }$	2 Hz	0.27
Case 2	0.37 m	0.255 m	$22.5^{\circ }$	3 Hz	0.6

**Table 2:** Detailed data on various propulsion performance metrics for caudal fins with differing flexibilities across two St numbers.

St	α	$P_{\mathrm{in}}$	$P_{o}$	η	$\bar{F_{T}}$	$\bar{C_{T}}$
0.27	∞	90.2001	41.4398	0.459421	16.5759	0.07749
	1	85.5195	39.4107	0.460839	15.7643	0.073697
	2	91.8757	42.4444	0.461976	16.9778	0.07937
	3	92.8040	42.8932	0.462191	17.1573	0.08021
0.6	∞	851.3552	180.8060	0.212374	72.3224	0.3381
	1	809.9895	170.1120	0.210018	68.0448	0.31811
	2	860.0029	181.8507	0.211454	72.7403	0.34006
	3	866.6092	183.3821	0.211609	73.3528	0.34292

## Data Availability

All data generated or analyzed during this study are included in this published article.
